# Glucagon‐Like Peptide‐1 Receptor Agonists in Cocaine Use Disorder: Clinical Observations and Underlying Mechanisms

**DOI:** 10.1111/fcp.70103

**Published:** 2026-06-30

**Authors:** Céline Eiden, Ghjulia Chautard, Anne Batisse, Aurélie Aquizerate, Amandine Luquiens, Hélène Donnadieu, Jean‐Luc Faillie, Hélène Peyriere

**Affiliations:** ^1^ Addictovigilance Centre of Montpellier, Département de Pharmacologie Médicale et Toxicologie CHU Montpellier Montpellier France; ^2^ Addictovigilance Centre of Paris, AP‐HP, Nord Université Paris Cité Paris France; ^3^ Addictovigilance Centre of Nantes, Nantes Université, CHU Nantes, Service de Pharmacologie Clinique Nantes France; ^4^ Department of Addictology CHU Nîmes, Université Montpellier Nîmes France; ^5^ Department of Addictology CHU Montpellier Montpellier France; ^6^ Centre de Pharmacovigilance, Département de Pharmacologie Médicale et Toxicologie CHU Montpellier Montpellier France

**Keywords:** addictovigilance, cocaine‐related disorders, dopaminergic pathways, glucagon‐like peptide‐1 receptor agonists, pharmacovigilance

## Abstract

**Background:**

Cocaine use disorder (CUD) remains a major public health concern, characterized by high relapse rates and the absence of approved pharmacological treatments. Glucagon‐like peptide‐1 receptor agonists (GLP‐1RA), widely prescribed for metabolic disorders, modulate mesolimbic reward pathways and have been proposed as potential modulators of addictive behaviors.

**Objectives:**

To describe clinical observations of GLP‐1RA exposure in individuals with CUD identified through the French national vigilance system and to contextualize these findings through a structured narrative review of preclinical and clinical evidence.

**Methods:**

Cases involving GLP‐1RA exposure in individuals with ongoing cocaine use were identified through the French national vigilance system and described. A structured literature search (MEDLINE, Embase, Google Scholar) identified original preclinical and clinical studies examining the effects of GLP‐1 receptor activation on cocaine‐related behavioral and neurobiological outcomes.

**Results:**

Two cases involving patient‐initiated off‐label GLP‐1RA use motivated by perceived anti‐craving effects were identified through the vigilance system. The literature synthesis included 22 original publications (17 preclinical and 5 human studies). Across experimental models, GLP‐1 receptor activation reduced cocaine self‐administration, conditioned reward, and relapse‐like behaviors. These effects were associated with the attenuation of mesolimbic dopamine signaling, including reduced cocaine‐evoked dopamine release and modulation of dopamine transporter function. Human evidence remains limited and heterogeneous.

**Conclusion:**

GLP‐1 receptor signaling represents a biologically plausible target in CUD. However, current clinical evidence remains preliminary. Patient‐driven observations underscore the need for controlled clinical trials and continued vigilance monitoring

**Abbreviations**
5‐HT_2C_
: 5‐hydroxytryptamine receptor 2C
5‐HT_3_
: 5‐hydroxytryptamine receptor 3
ANPV: National Application of Pharmacovigilance Database
ANSM: French National Agency for Medicines and Health Products Safety
CUD: cocaine use disorder
DAT: dopamine transporter
dLS: dorsal lateral septum
DREADDs: designer receptors exclusively activated by designer drugs
GABA: gamma‐aminobutyric acid
GLP‐1: glucagon‐like peptide‐1
GLP‐1RA: glucagon‐like peptide‐1 receptor agonist
IP: intraperitoneal
IV: intravenous
LDTg: laterodorsal tegmental nucleus
LS: lateral septum
NAc: nucleus accumbens
NTS: nucleus tractus solitarius
PFC: prefrontal cortex
PPG: preproglucagon
VTA: ventral tegmental area

## 1 Introduction

Cocaine use disorder (CUD) remains a major public health concern worldwide, associated with substantial morbidity, mortality, and social burden [1]. To date, no pharmacological treatment has received regulatory approval for the management of cocaine dependence, and relapse rates remain high even in individuals engaged in structured psychosocial interventions [2].

Cocaine addiction is primarily driven by maladaptive activation of the mesolimbic reward system, in which dopaminergic projections from the ventral tegmental area (VTA) to the nucleus accumbens (NAc) play a central role [3]. By blocking the dopamine transporter, cocaine induces a rapid and marked increase in extracellular dopamine levels within the NAc, reinforcing drug‐taking behavior and strengthening associative learning processes related to drug‐associated cues. Repeated cocaine exposure further leads to persistent neuroadaptations within this circuitry, contributing to craving, loss of control, and vulnerability to relapse [4].

Despite this well‐characterized dopaminergic mechanism, systematic reviews and meta‐analyses of pharmacotherapy trials for CUD have consistently failed to identify any medication capable of improving abstinence, reducing cocaine consumption, or enhancing treatment retention, and have highlighted the absence of approved pharmacological options in this field [5, 6]. Psychosocial interventions therefore remain the cornerstone of care, highlighting the need to develop new pharmacological strategies to indirectly modulate the reward mechanisms underlying reinforcement, craving, and relapse associated with cocaine use. These limitations have prompted growing interest in neuromodulatory systems capable of fine‐tuning mesolimbic reward circuits without directly interfering with dopaminergic transmission.

Among emerging neuromodulatory systems implicated in the regulation of reward‐related behaviors, glucagon‐like peptide‐1 (GLP‐1) signaling has gained increasing attention beyond its established role in glucose homeostasis and energy balance. GLP‐1 is a gut–brain peptide produced both peripherally by intestinal L cells and centrally by preproglucagon (PPG) neurons located in the nucleus tractus solitarius (NTS), with widespread projections to brain regions involved in motivation, reward processing, and executive control [7, 8]. GLP‐1 receptors are expressed throughout the mesolimbic system, including the VTA and NAc, positioning this pathway as a potential modulator of dopaminergic signaling relevant to substance use disorders [9, 10].

Beyond these mechanistic considerations, the rapidly expanding use of GLP‐1 receptor agonists (GLP‐1RA) for weight loss indication has markedly increased their availability and visibility. This expansion has been accompanied by emerging patterns of off‐label and nonmedical use, particularly for weight loss in individuals without approved indications, as documented in a recent nationwide pharmacoepidemiological study [11].

In this context of increasing availability and emerging misuse, individuals with substance use disorders (e.g., alcohol, opioids, stimulants, nicotine) or behavioral disorders involving dysregulated reward processing, such as binge eating, may represent a particularly vulnerable population in which off‐label exploration of GLP‐1RA could occur [12]. However, such practice remains poorly documented.

The present article aims to describe cases of patient‐initiated or off‐label GLP‐1RA use in individuals with CUD identified through the French pharmacovigilance and addictovigilance system, and to contextualize these observations within a structured narrative review of preclinical and clinical evidence regarding GLP‐1 receptor signaling in cocaine‐related behaviors.

## 2 Materials and Methods

### 2.1 Addictovigilance and Pharmacovigilance Data Sources

Spontaneous reports involving GLP‐1RA in individuals using cocaine were identified through the French National Application of Pharmacovigilance Database (ANPV), the national electronic reporting system coordinated by the French National Agency for Medicines and Health Products Safety (ANSM). The ANPV includes dedicated modules for pharmacovigilance and addictovigilance surveillance.

The pharmacovigilance module collects reports of suspected adverse drug reactions, medication errors, misuse, abuse, and off‐label use when associated with safety concerns. The addictovigilance module specifically monitors abuse, dependence, diversion, and problematic use patterns involving psychoactive substances, including prescription medications.

Reports are primarily submitted by healthcare professionals (physicians, pharmacists, addiction specialists), although patient reporting is also possible.

A complementary search was conducted in VigiBase, the World Health Organization (WHO) global database of individual case safety reports (ICSRs), maintained by the Uppsala Monitoring Centre. VigiBase compiles reports submitted by national pharmacovigilance centers participating in the WHO Programme for International Drug Monitoring. Reports may include adverse reactions, misuse, medication errors, and off‐label use when reported as safety concerns.

### 2.2 Case Identification and Search Strategy

The search strategy combined terms related to GLP‐1 receptor agonists (“GLP‐1”, “GLP‐1 receptor agonist”, individual molecule names) with the term “cocaine”. Cases were included if they described intentional or off‐label GLP‐1RA use in individuals reporting ongoing cocaine use or CUD.

For each case, the following data were extracted when available: patient demographics (age, sex), history of psychoactive substance use, characteristics of GLP‐1RA exposure (molecule, dose, treatment context), patterns of use (intentional misuse, off‐label use, procurement), associated clinical manifestations and seriousness criteria according to WHO definitions (e.g., hospitalization, life‐threatening condition, disability). All cases were anonymized in accordance with French regulatory requirements for pharmacovigilance and addictovigilance data collection and analysis.

### 2.3 Literature Search and Study Selection

A structured literature search was conducted using MEDLINE (via PubMed), Embase, and Google Scholar to identify relevant publications. Searches were performed from database inception to January 15, 2026. The review was conducted in accordance with principles of transparent reporting for narrative reviews, including explicit description of the search strategy, inclusion criteria, and study selection process, in line with SANRA recommendations [13].

PubMed search string (“Glucagon‐Like Peptide 1”[MeSH Terms] OR GLP‐1[tiab] OR “glucagon‐like peptide‐1”[tiab] OR “GLP‐1 receptor agonist”[tiab] OR exenatide[tiab] OR liraglutide[tiab] OR semaglutide[tiab] OR dulaglutide[tiab] OR lixisenatide[tiab] OR tirzepatide[tiab]) AND (“Cocaine”[MeSH Terms] OR cocaine[tiab] OR “cocaine use disorder”[tiab] OR “cocaine dependence”[tiab] OR “cocaine craving”[tiab] OR addiction[tiab]) NOT (“cocaine‐ and amphetamine‐regulated transcript”[tiab] OR CART[tiab]).

Equivalent combinations were adapted for Embase and Google Scholar.

No restriction was applied regarding the initial therapeutic indication of GLP‐1RA. Eligible publications included original studies reporting:

- clinical observations of GLP‐1RA exposure in individuals with CUD,
- secondary or incidental changes in cocaine‐related outcomes in patients treated with GLP‐1RA for approved metabolic indications,
- or experimental investigations of GLP‐1 receptor signaling in cocaine‐related behavioral or neurobiological paradigms.

Both preclinical and clinical studies were eligible. Review articles, editorials, commentaries, and studies not directly addressing cocaine‐related outcomes were excluded from the core analysis but could be cited for contextual background.

The PubMed search yielded 58 records. No additional eligible records were identified in Embase. One additional relevant original study was identified through supplementary searches using Google Scholar, resulting in a total of 59 records. After title and abstract screening, 37 articles were excluded (reviews, nonoriginal publications, or studies not directly addressing cocaine‐related outcomes). The remaining 22 studies were assessed in full and included in the narrative synthesis. Study selection was performed independently by three authors, with discrepancies resolved through discussion. For preclinical studies, extracted items included the animal model, GLP‐1 signaling manipulation, cocaine‐related paradigm and/or endpoint, main behavioral and neurobiological findings and, when available, controls for nonspecific effects. For human studies, we performed a structured qualitative assessment of study quality and main risk‐of‐bias considerations, including study design, sample size, control condition, randomization/blinding, treatment duration, outcome assessment, and key limitations.

## 3 Results

### 3.1 Clinical Observations From Addictovigilance and Pharmacovigilance Data Sources

Two cases were identified through the ANPV. The complementary search in VigiBase did not identify additional international cases. Both cases involved patient‐initiated, off‐label GLP‐1RA use motivated by weight loss and/or a perceived anti‐addiction effect. They are summarized in Table 1, together with the two published clinical case reports identified through the literature search.

**TABLE 1:** Original vigilance cases and published clinical case reports of GLP‐1 receptor agonist exposure in individuals with cocaine use disorder.

Source	Patient characteristics	Name of GLP‐1RA context of use	BMI/metabolic status	Concomitant medications	Cocaine use changes after GLP‐1RA initiation	Alcohol changes after GLP‐1RA initiation	Safety
French vigilance case 1	Male, age NR, regular intranasal cocaine use.	Semaglutide; initially prescribed, then continued as self‐medication via online supply.	Obesity (> 150 kg).	Antidepressant treatment and alprazolam.	Marked reduction in cocaine craving reported.	Stopped drinking and experiencing a reduced desire to drink.	No AE reported.
French vigilance case 2	Middle‐aged male, 25‐year history of intranasal CUD; cocaine use up to 4 g per episode.	Tirzepatide 5 mg once weekly; obtained via teleconsultation; intentional use for weight loss and perceived anti‐addiction effect.	History of adolescent obesity with occasional hyperphagia; no current BMI documented.	Bilastine 20 mg/day; azelastine/fluticasone nasal spray; nalmefene 18 mg/day; ongoing addiction follow‐up.	Reduced cocaine use frequency and amount, craving not documented.	Reduced alcohol intake per occasion, persistent craving.	No AE reported.
Romeo 2025	54‐year‐old male; 15‐year history of CUD.	Semaglutide; 0.25 mg/week titrated to 1.0 mg/week over 12 weeks; supervised treatment.	Obesity BMI 35.4 kg/m^2^ at baseline, decreasing to 31.0 kg/m^2^ after 12 weeks.	Supervised medical treatment; other medications not specified.	58.9% reduction in CCQ‐Brief over 12 weeks.	NR.	No AE reported.
Sileoni et al. 2025	33‐year‐old female; severe CUD; borderline personality disorder.	Oral semaglutide 3 mg daily; supervised treatment.	Insulin resistance and diabetes mellitus.	Psychopharmacological treatment maintained.	> 75% reduction in CCQ subscales over 3 months.	NR.	No AE reported.

*Note:* The two “French vigilance” cases were identified through the French pharmacovigilance/addictovigilance system. Romeo [14] and Sileoni et al. [15] were previously published case reports identified through the literature review.

Abbreviations: CCQ‐Brief, Cocaine Craving Questionnaire–Brief; CUD, cocaine use disorder; GLP‐1RA, glucagon‐like peptide‐1 receptor agonist; NR, not reported.

### 3.2 Literature Findings

**FIGURE 1:**
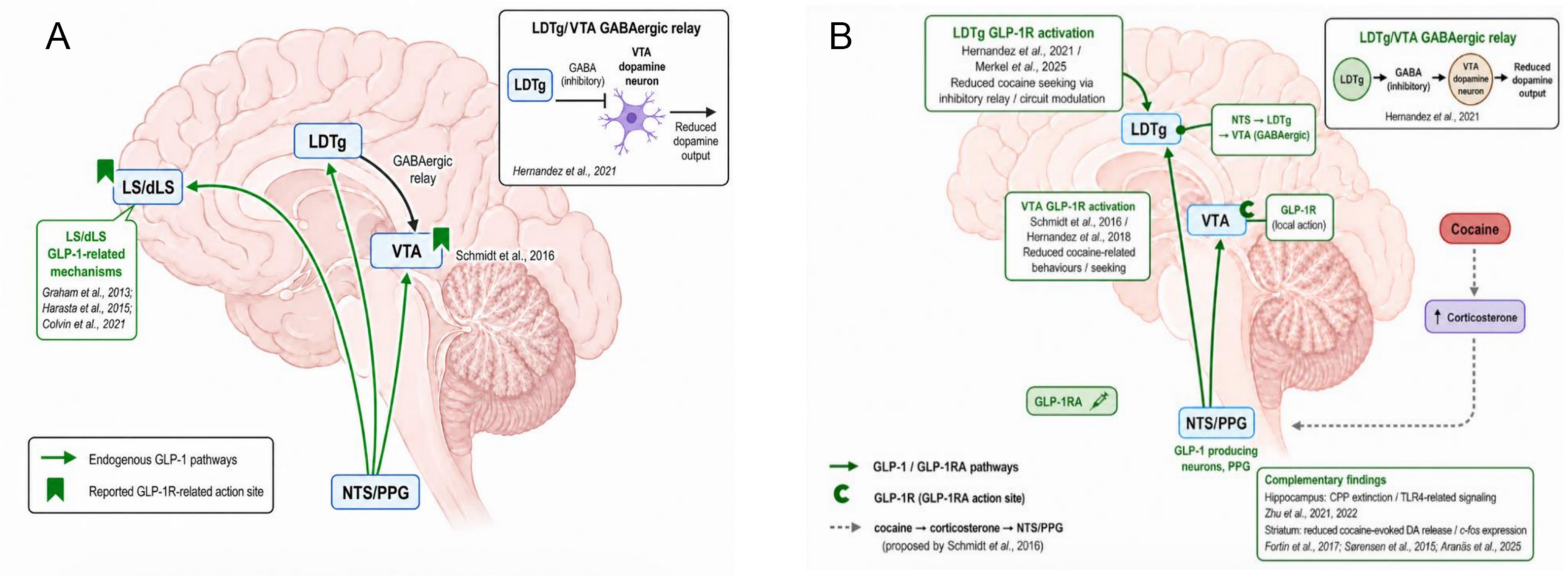
Flow chart of study selection. Records were identified through PubMed, Google Scholar, and Embase. After title and abstract screening, 22 full‐text articles were assessed for eligibility and all were included in the narrative synthesis. The final selection comprised 17 preclinical studies and 5 human studies.

A total of 22 original publications met the inclusion criteria and were included in this narrative synthesis [14, 15, 16, 17, 18, 19, 20, 21, 22, 23, 24, 25, 26, 27, 28, 29, 30, 31, 32, 33, 34, 35]. The study selection process is summarized in Figure 1. Of these, 17 were preclinical investigations conducted in animal models and were summarized in Table 2 [16, 17, 18, 19, 20, 21, 22, 23, 24, 25, 26, 27, 28, 29, 30, 31, 32]. The remaining five involved human participants: three experimental or interventional human studies, summarized in Table 3 [33, 34, 35], and two previously published clinical case reports, summarized together with the two original vigilance cases in Table 1 [14, 15].

**TABLE 2:** Preclinical studies investigating GLP‐1 receptor signaling in cocaine‐related outcomes. Studies were grouped according to the main brain region involved in the GLP‐1–related manipulation or downstream cocaine‐related outcome.

Study author, year	Model‐population	Experimental manipulation of GLP‐1 signaling	Cocaine‐related paradigm/endpoint	Main findings	Controls for nonspecific effects
VTA‐related studies					
Schmidt et al. 2016	Male Sprague–Dawley rats.	Intra‐VTA exendin‐4 injection (0.005–0.05 μg/kg); intraventricular corticosterone; GLP‐1R knockdown.	Cocaine self‐administration; endogenous GLP‐1 response.	Activation of GLP‐1R in the VTA reduces cocaine self‐administration. Cocaine activates GLP‐1 neurons in the NTS (probably via corticosterone). Knockdown of GLP‐1R in the VTA increases cocaine consumption.	Sucrose self‐administration.
Hernandez et al. 2018	Male Sprague–Dawley rats.	Systemic administration of exendin‐4 (fluoro‐Ex‐4; 0.01–3.0 μg/kg, IP) Intra‐VTA microinjection of exendin‐4 (0.005–0.05 μg/100 nL) intra‐VTA GLP‐1 receptor antagonism.	Cocaine self‐administration; extinction/relapse‐like cocaine seeking; endogenous GLP‐1 response.	Systemic and intra‐VTA exendin‐4 reduced cocaine reinstatement; effects were blocked by intra‐VTA GLP‐1R antagonism, confirming a VTA‐dependent mechanism. Extinction decreased NTS PPG mRNA expression.	Food intake; meal pattern; body weight; sucrose reinstatement.
Colvin et al. 2021	Male Sprague–Dawley rats.	Intra‐VTA exendine‐4 injection (0.05–0.5 μg/kg).	Psychostimulant‐induced alcohol intake, including cocaine‐induced alcohol intake.	Exendin‐4 administration in the VTA inhibits the increase in alcohol intake induced by psychostimulant (included cocaine) and blocks ghrelin‐mediated potentiation of this effect.	Food/water available; no specific satiety control.
Merkel et al. 2025	Sprague–Dawley rats and transgenic rats (Long–Evans Gad1‐Cre and Sprague–Dawley Th‐Cre).	DREADD activation of NTS → VTA circuit; systemic exendin‐4 (2.0 μg/kg).	Cocaine self‐administration; cocaine seeking; endogenous GLP‐1 response; GABA/dopamine neuron activity.	Cocaine self‐administration decreased plasma GLP‐1 levels. Activation of the endogenous NTS → VTA circuit reduces cocaine seeking. Exendine‐4 increased VTA GABA neurons activity and decreased dopamine neuronal activity.	Chow intake; body weight; mCherry/CNO control.
NAc‐related studies					
Fortin et al. 2017	Male Sprague–Dawley rats.	Intraventricular exendin‐4, (0.15 μg/kg).	Cocaine‐evoked dopamine signaling; dopamine reuptake.	Exendin‐4 suppressed cocaine‐evoked phasic dopamine signaling selectively in the NAc core, with no effect in the shell and no significant impact on dopamine reuptake.	Not applicable.
Hernandez et al. 2019	Male Sprague–Dawley rats.	Intra‐NAc exendin‐4 injection (core and shell); 0.005 and 0.05 μg/kg. Systemic fluoro‐exendin‐4 (0.1 and 0.2 μg/kg).	Cocaine self‐administration; extinction/relapse‐like cocaine seeking; neuronal excitability.	Exendin‐4 reduced reinstatement of cocaine seeking. In cocaine‐experienced rats, exendin‐4 increased intrinsic excitability of medium spiny neurons.	Body weight; chow intake; meal frequency/size; sucrose seeking.
Zhu et al. 2022	Male C57BL/6J mice.	Systemic exendin‐4 (2.0 μg/kg, IP) administration.	Conditioned place preference; reinstatement; cocaine‐induced hyperlocomotion; neuroinflammatory markers.	Exendin‐4 attenuated cocaine‐ and stress‐induced reinstatement of CPP and reduced cocaine‐associated hyperlocomotion. Reduced cocaine‐induced NF‐κB p65 expression in the NAc.	Locomotor activity; no satiety control.
Aranäs et al. 2025	Male Sprague–Dawley rats; male NMRI mice (microdialysis).	Subcutaneous semaglutide (0.026 and 0.039 mg/kg) injection.	Cocaine self‐administration; progressive‐ratio responding; reinstatement; cocaine‐evoked dopamine signaling.	Semaglutide reduced cocaine self‐administration, motivation (breakpoint), and reinstatement (relapse). It attenuated cocaine‐induced dopamine elevation in the NAc without evidence of aversive effects.	Kaolin intake; locomotor activity; food/body weight/water intake.
LS‐related studies					
Harasta et al. 2015	GLP‐1R knockout male mice and wild‐type controls.	Genetic deletion of the GLP‐1 receptor; viral re‐expression.	Cocaine‐induced locomotion; conditioned place preference.	GLP‐1R‐deficient mice exhibited increased cocaine‐induced locomotion and conditioned place preference. Selective re‐expression of GLP‐1R in the dLS normalizes these behaviors.	Locomotion/stereotypy; anxiety‐like behavior; no food/satiety control.
Reddy et al. 2016	Male NMRI mice; male C57BL/6 mice; male Sprague–Dawley rats.	GLP‐1 (ex vivo); exendin‐4 (in vivo, 2.4 μg/kg).	Cocaine‐induced locomotion; cocaine‐evoked dopamine signaling; dopamine homeostasis/DAT function; lipid signaling.	GLP‐1R activation reduced arachidonic acid and 2‐arachidonoylglycerol levels, leading to increase dopamine transporter (DAT) surface expression and function. This mechanism attenuated cocaine‐induced dopamine elevation.	Not applicable.
Hernandez et al. 2021	Sprague–Dawley rats; GAD1‐Cre transgenic rats.	Intra‐LDTg exendin‐4 (0.005 and 0.025 μg/kg); viral knockdown; DREADDs.	Cocaine self‐administration; extinction/reinstatement; GABAergic circuit activity.	Activation of GLP‐1R in the LDTg reduced cocaine seeking. Receptors were localized to GABAergic neurons. Activation of the LDTg → VTA GABAergic pathway was sufficient to decrease drug‐seeking behavior.	Sucrose seeking; food intake; body weight.
Striatum‐related studies					
Egecioglu et al. 2013	Male NMRI mice.	Systemic exendin‐4 (2.4 μg/kg, IP); cocaine self‐administration (10, 30, and 100 μg/kg).	Cocaine‐induced hyperlocomotion; conditioned place preference; cocaine‐evoked dopamine signaling.	Exendin‐4 reduced cocaine‐induced hyperlocomotion, attenuated dopamine release in the NAc, and block conditioned place preference.	Ex4 alone locomotion/dopamine; prior CPP control; no food/satiety control.
Sorensen et al. 2015	Male NMRI mice; male C57Bl/6 mice.	Exendin‐4 (0.3–30 μg/kg, IP).	Cocaine self‐administration; cocaine‐induced hyperlocomotion; striatal c‐fos expression; cocaine‐evoked dopamine signaling.	Exendin‐4 reduced acute and chronic self‐administration, cocaine‐induced hyperlocomotion, striatal c‐fos expression, and cocaine‐evoked dopamine release.	Basal locomotion; saline/inactive responding; no food/satiety control.
Hippocampus‐related studies					
Zhu et al. 2021	Male C57BL/6 mice.	Systemic exendin‐4 (0.1–100 μg/kg, IP) during conditioning, testing, or post‐extinction.	Conditioned place preference; extinction/reinstatement; neuroinflammatory markers.	Exendin‐4 reduced acquisition and expression of cocaine CPP, facilitated extinction, and attenuated reinstatement. Reduced hippocampal TLR4/TNF‐α/IL‐1β.	Locomotor activity; no natural reward/satiety control.
Zhu et al. 2021	Male C57BL/6 mice.	Systemic exendin‐4 (1 μg/kg, IP) post‐extinction (immediate vs. 6 h delay); hippocampal TLR4 measurement.	Conditioned place preference; extinction/reinstatement; neuroinflammatory markers.	Repeated exendin‐4 administered immediately after extinction sessions facilitated extinction of cocaine‐induced CPP and attenuated cocaine‐primed reinstatement, whereas delayed administration was ineffective. Behavioral effects were associated with reduced hippocampal TLR4 expression, suggesting modulation of neuroinflammatory signaling during extinction memory consolidation.	Locomotor activity; no natural reward/satiety control.
Systemic GLP‐1R activation‐related studies					
Graham et al. 2013	Male mice.	Systemic exendin‐4 pretreatment (10–100 μg/kg).	Conditioned place preference; cocaine‐induced locomotion; conditioned aversion.	Exendin‐4 pretreatment dose‐dependently attenuated cocaine‐induced CPP without affecting locomotor stimulation or inducing aversion, indicating reduced cocaine reward via GLP‐1R activation.	GLP‐1R KO comparison; no food/satiety control.
You et al. 2019	Long–Evans rats.	Measurement of endogenous circulating GLP‐1.	Cocaine self‐administration; cocaine availability anticipation; endogenous GLP‐1/metabolic hormone response.	Cocaine self‐administration and anticipation of cocaine availability were associated with increased circulating GLP‐1 levels.	Not applicable.

*Note:* The term “cocaine‐related paradigm/endpoint” refers to the experimental procedure used to establish or assess cocaine‐related behavior, such as self‐administration, conditioned place preference, extinction/reinstatement, or acute cocaine challenge, and the main behavioral or neurobiological endpoint measured.

**TABLE 3:** Published human studies investigating GLP‐1 receptor signaling in cocaine exposure and cocaine use disorder.

Study author, year	Population	GLP‐1 intervention/measure	Main findings	Study quality/main risk‐of‐bias considerations
Bouhlal et al. 2017	Experienced cocaine users (non–treatment‐seeking), experimental intravenous cocaine administration (25 mg).	Measurement of endogenous GLP‐1 and peptide YY.	Acute IV cocaine decreased circulating GLP‐1 and peptide YY concentrations.	Exploratory controlled human laboratory study; very small sample (*n* = 8); standardized IV cocaine exposure and hormone assessment; limited by absence of placebo cocaine condition and causal inference.
Angarita et al. 2021	Adults with cocaine use disorder (non–treatment‐seeking); experimental human laboratory study using IV cocaine self‐administration.	Acute subcutaneous exenatide (5 μg/kg).	Exenatide did not reduce cocaine self‐administration or subjective effects (euphoria/craving). Cocaine exposure reduced plasma GLP‐1 levels.	Randomized double‐blind placebo‐controlled crossover study; small sample (*n* = 13); standardized cocaine self‐administration and subjective outcomes; limited by acute single low‐dose exenatide exposure and non–treatment‐seeking population.
Yammine et al. 2023	Adults with cocaine use disorder; open‐label outpatient case series. Cocaine use assessed via self‐report and urine drug screens.	Extended‐release exenatide (2 mg weekly for 6 weeks).	One participant achieved sustained abstinence with reduced craving, while two participants continued cocaine use. Treatment was feasible and well tolerated.	Open‐label case series; very small sample (*n* = 3); repeated extended‐release exenatide with feasibility/safety assessment; limited by the absence of a control group, no blinding/randomization, concomitant counseling, and heterogeneous outcomes.

#### 3.2.1 Preclinical Mechanistic Studies

Preclinical studies were grouped according to the primary brain region in which GLP‐1 receptor signaling was experimentally manipulated or assessed, as defined by the site of pharmacological, genetic, or circuit‐level intervention.

Across experimental models, activation of GLP‐1 receptor signaling consistently reduced cocaine‐related behaviors, including cocaine intake, motivation for cocaine, conditioned place preference, and relapse‐like cocaine seeking. These effects were observed following both systemic and region‐specific GLP‐1 receptor agonist administration, supporting an inhibitory influence of GLP‐1 signaling on cocaine reinforcement and relapse‐related outcomes [16, 17, 19, 21, 22, 23, 26, 27, 28, 29, 30, 31]. Nevertheless, none of the included studies directly evaluated aversive withdrawal‐related symptoms as a primary outcome.

At the circuit level, VTA‐related studies provide evidence for a local GLP‐1R–dependent mechanism. Intra‐VTA GLP‐1R activation reduced cocaine self‐administration or reinstatement, whereas VTA GLP‐1R knockdown increased cocaine intake, and intra‐VTA GLP‐1R antagonism blocked the effects of systemic exendin‐4. In addition, activation of NTS‐derived GLP‐1 projections to the VTA and LDTg reduced cocaine seeking, while LDTg GLP‐1R activation recruited a GABAergic projection to the VTA and decreased dopamine neuron activity, whereas several NAc findings reflect downstream effects rather than direct local NAc inhibition [19, 20, 21, 23, 26]. Together, these studies support a role for GLP‐1 signaling in regulating VTA dopamine neuron excitability through local and upstream inhibitory mechanisms.

At the neurobiological level, GLP‐1 receptor activation attenuated cocaine‐evoked dopaminergic signaling within the mesolimbic system. Several studies demonstrated suppression of cocaine‐induced dopamine release in the NAc, particularly affecting phasic dopamine signaling associated with reinforcement and reward prediction [20, 23, 27, 28, 31].

Beyond mesolimbic dopamine neuron modulation, GLP‐1 receptor signaling was also shown to regulate dopamine homeostasis through mechanisms distinct from direct modulation of mesolimbic dopamine neuron activity. Activation of GLP‐1 receptors within the lateral septum (LS) increased dopamine transporter surface expression and normalized dopamine clearance, thereby limiting cocaine‐induced dopamine elevations through mechanisms involving lipid‐mediated signaling pathways [22, 24, 25, 29, 30].

Hippocampal studies suggested that GLP‐1RA may influence conditioned place preference extinction and cocaine‐associated TLR4‐related inflammatory signaling [29, 30]. Striatal findings indicated modulation of cocaine‐evoked dopamine release and c‐fos expression [27, 28]. In addition, several studies implicated neuroinflammatory mechanisms in GLP‐1–mediated effects [22, 29, 30]. In hippocampal and NAc models, exendin‐4 reduced cocaine‐associated changes in TLR4‐related inflammatory markers and NF‐κB signaling. These findings suggest that GLP‐1RA may influence cocaine‐associated neuroimmune responses, although they do not establish that these changes causally mediate behavioral effects.

**FIGURE 2:**
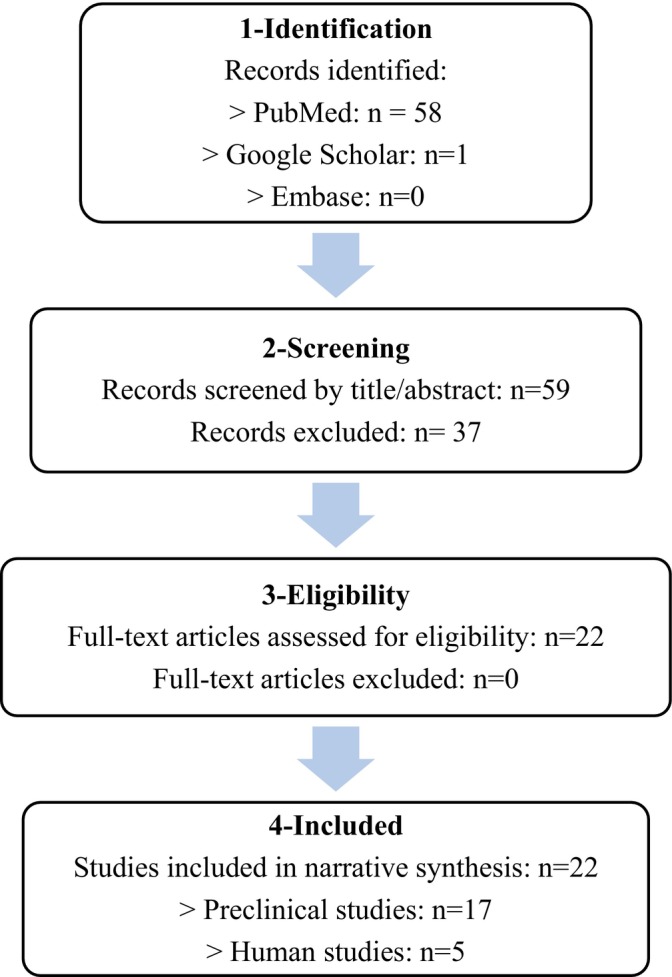
Proposed GLP‐1R–mediated circuits and mechanisms involved in cocaine‐related behaviors.

Collectively, these findings indicate that GLP‐1RA modulates cocaine‐related behaviors through complementary mechanisms involving dopamine neuron excitability, phasic dopamine release, dopamine transporter regulation, and neuroimmune signaling. The principal GLP‐1–dependent circuits involved in cocaine‐related behaviors are summarized in Figure 2.

#### 3.2.2 Human Studies

Five publications investigated GLP‐1RA or GLP‐1 signaling in individuals with cocaine exposure or CUD [14, 15, 33, 34, 35].

Three experimental human studies examined GLP‐1 signaling in the context of cocaine exposure or treatment [33, 34, 35]. In one study, acute IV cocaine administration was associated with decreased circulating GLP‐1 levels in experienced users [33]. In a randomized exploratory study conducted in non–treatment‐seeking individuals with CUD, acute subcutaneous exenatide did not reduce cocaine self‐administration or cocaine‐related subjective effects, whereas cocaine exposure reduced plasma GLP‐1, insulin, and amylin concentrations [34].

In a small open‐label pilot study, 6 weeks of once‐weekly extended‐release exenatide was feasible and well tolerated; one of three participants achieved sustained abstinence with reduced craving, whereas the others continued cocaine use during follow‐up [35].

Two additional case reports published in the literature reported clinical improvement following semaglutide initiation for metabolic indications [14, 15]. Romeo [14] reported a 54‐year‐old obese man (BMI 35.4 kg/m^2^) with a 15‐year history of CUD who received semaglutide within a supervised medical framework for obesity, in accordance with approved indications. The treatment began with a weekly dose of 0.25 mg, gradually increased to 1.0 mg by week 8, following standard titration protocols to minimize gastrointestinal side effects. Over a 12‐week treatment period, the patient exhibited a 58.9% reduction in cocaine craving assessed using the Cocaine Craving Questionnaire–Brief (CCQ‐Brief score 5.6 to 2.3) and a 12.4% reduction in body weight (BMI 31.0 kg/m^2^). No serious adverse effects were reported.

Sileoni et al. [15] described a 33‐year‐old woman with severe CUD and borderline personality disorder, hospitalized in a psychiatric setting and presenting with insulin resistance and diabetes mellitus. Semaglutide was initiated for metabolic indications while psychopharmacological treatment was maintained. Over a 3‐month follow‐up, marked reductions in cocaine craving were observed, with Cocaine Craving Questionnaire subscale scores decreasing by more than 75%. Impulsivity (BIS‐11) declined substantially (from 80 to 33), binge‐eating behaviors remitted, and anxiety symptoms improved. No adverse effects were reported.

## 4 Discussion

This study describes the first identified cases of patient‐initiated, off‐label use of GLP‐1RA in individuals with CUD and situates these observations within a mechanistic framework derived from experimental studies on GLP‐1 signaling and cocaine‐related behaviors.

In the two cases identified through pharmacovigilance/addictovigilance systems, GLP‐1RA were used off‐label by patients with the explicit intention of reducing cocaine craving. This self‐directed therapeutic use occurred despite the absence of regulatory approval or robust clinical evidence supporting GLP‐1RA in CUD. Although such observations cannot establish efficacy, they highlight an emerging perception of GLP‐1RA as potential “anti‐addiction” agents and underscore the need for controlled clinical investigation.

From a public health perspective, these observations should be considered. The acquisition of GLP‐1RA outside any medical supervision, whether through informal channels, online sources, or teleconsultation, reflects the increasing availability and visibility of these medications.

In contrast to these cases, two supervised clinical case reports have described GLP‐1RA treatment in individuals receiving the medication within approved metabolic indications [14, 15]. Although standardized assessments of craving and behavioral symptoms were used and clinical improvements were reported, such case reports provide only low‐level clinical evidence. By design, these clinical observations lack control conditions and cannot exclude alternative explanations, including spontaneous remission, regression to the mean, expectancy effects, contextual influences related to structured medical supervision, concomitant medications, psychiatric or addiction care, and metabolic factors. Accordingly, changes in cocaine craving or use cannot be attributed solely to GLP‐1RA exposure.

Whether GLP‐1RA effects on cocaine‐related outcomes differ according to BMI or metabolic status remains unknown. Although obesity and metabolic disorders may plausibly modify response through shared reward‐related and metabolic pathways, available uncontrolled observations do not support the conclusion that obesity predicts a greater CUD‐attenuating effect.

Although in all cases, no serious adverse events were reported, the absence of acute toxicity should not be interpreted as evidence of overall safety, especially given the long duration of action of these compounds and their complex central and peripheral effects. Moreover, the persistence of potential effects on cocaine craving after discontinuation of GLP‐1RA remains unknown, raising questions about the durability of any observed benefit.

Ongoing pharmacovigilance assessments continue to monitor potential long‐term safety signals, including thyroid‐related risks. Recent comprehensive reviews suggest that early concerns regarding pancreatic and thyroid cancer have been attenuated by accumulating evidence. However, other safety considerations remain under active evaluation, including gallbladder and biliary disorders, perioperative aspiration risk, and weight regain following treatment discontinuation [36, 37, 38]. Earlier concerns regarding suicidal ideation and behavior have been re‐evaluated; a recent FDA review did not support an increased risk or causal association, leading the Agency to request removal of such warnings from the labelling of concerned GLP‐1RA medications [39]. Nevertheless, psychiatric monitoring remains clinically relevant in CUD populations because of their baseline psychiatric vulnerability and frequent comorbidities. The absence of additional international cases identified through VigiBase should be interpreted with caution. Spontaneous pharmacovigilance systems are primarily designed to detect suspected adverse drug reactions and safety concerns. Incidental clinical improvements in comorbid conditions, such as reductions in cocaine craving occurring during treatment prescribed for approved metabolic indications, do not constitute adverse events and are therefore unlikely to be systematically reported. Consequently, pharmacovigilance databases are structurally ill‐suited to detect potential therapeutic benefits in addiction unless associated with misuse, safety issues, or regulatory concerns.

The preclinical literature provides a coherent and internally consistent mechanistic framework supporting a role for GLP‐1 signaling in the modulation of cocaine reinforcement, motivation, and relapse‐like behaviors [16, 27, 28]. Across multiple animal models and experimental paradigms, GLP‐1 receptor activation reliably attenuated cocaine taking, seeking, and cue‐ or drug‐induced reinstatement [16, 21, 28]. These behavioral effects were associated with reductions in mesolimbic dopaminergic activity, a core neurobiological substrate of cocaine addiction [16, 21, 27].

Importantly, the convergence of findings across species, experimental designs, and pharmacological compounds enhances their biological plausibility and supports cautious translational consideration. From a translational perspective, systemic GLP‐1RA studies are particularly relevant because local intracranial administration is not feasible in humans. However, because GLP‐1 signaling modulates appetitive and reward‐related behaviors more broadly, reduced cocaine‐related responses may not be entirely cocaine‐specific. Some studies nevertheless included controls arguing against a purely nonspecific suppression of appetitive behavior: Schmidt et al. found that intra‐VTA exendin‐4 reduced cocaine but not sucrose self‐administration; Hernandez et al. showed that low‐dose systemic fluoro‐exendin‐4 reduced cocaine seeking without affecting body weight or meal patterns; and Hernandez et al. later reported that low‐dose intra‐LDTg exendin‐4 attenuated cocaine seeking without altering sucrose seeking, food intake, or body weight. More recently, Aranäs et al. reported no increase in kaolin intake and no suppression of locomotor activity with semaglutide, although reductions in food intake, body weight, and water intake indicate that metabolic effects remain a potential confounder. These findings support some degree of selectivity in specific paradigms, while underscoring the need to systematically assess natural reward, satiety, and malaise‐related outcomes in future studies.

Notably, GLP‐1–mediated effects on cocaine‐related behaviors are not restricted to a single neurobiological pathway. Experimental data indicate involvement of both classical mesolimbic circuits, particularly modulation of VTA to NAc dopamine signaling, and noncanonical mechanisms, including septal regulation of DAT function and lipid‐mediated signaling processes [21, 23, 26]. In addition, VTA data support a local GLP‐1R–dependent inhibitory mechanism, likely involving GABAergic control of dopamine neurons, whereas several NAc findings reflect downstream effects rather than direct local NAc inhibition [19, 26]. Hippocampal and striatal regions are highly relevant to contextual, conditioned, stress‐related, and motivational aspects of cocaine‐related behaviors. In the reviewed studies, hippocampal data mainly suggest that GLP‐1RA may influence conditioned place preference extinction, whereas striatal findings indicate modulation of cocaine‐evoked dopamine release and c‐fos expression.

Available data also suggest that GLP‐1RA may modulate cocaine‐associated neuroimmune responses, although their causal contribution to behavioral effects remains uncertain.

Such multimodal regulation of dopamine homeostasis may be particularly relevant in CUD, where repeated exposure induces complex and region‐specific neuroadaptations that extend beyond simple hyperdopaminergic states.

Beyond receptor localization and circuit‐level mechanisms, emerging data indicate that the temporal dynamics of GLP‐1 receptor activation may also be critical. Aranäs et al. [23] reported that semaglutide, a long‐acting GLP‐1RA, suppressed cocaine self‐administration, cocaine seeking, and cocaine‐evoked dopamine release in the NAc. Sustained receptor activation may allow more stable modulation of mesolimbic dopaminergic signaling compared to shorter‐acting compounds, potentially limiting fluctuations in dopamine neuron excitability associated with cocaine exposure.

This pharmacokinetic distinction may partly contribute to the discrepancy between preclinical findings and human experimental data. In particular, the absence of effect following acute exenatide administration in a controlled human laboratory study may reflect insufficient duration of receptor engagement, whereas repeated or long‐acting GLP‐1RA exposure could theoretically produce more substantial behavioral effects.

Clinical evidence supporting the use of GLP‐1RA in CUD remains limited. To date, it includes a human laboratory study conducted in non–treatment‐seeking individuals with CUD (*n* = 13), which evaluated the effects of a single acute subcutaneous dose of exenatide (5 μg) on IV cocaine self‐administration. This study did not demonstrate reductions in cocaine infusions or subjective effects. Although both exenatide and cocaine independently decreased circulating GLP‐1 and insulin levels, these neuroendocrine changes were not associated with measurable modifications in cocaine self‐administration behavior [34].

These findings are consistent with an earlier exploratory human laboratory study showing that acute IV cocaine decreased circulating GLP‐1 and PYY, with trends toward reduced insulin and amylin, suggesting that cocaine may acutely disrupt short‐term metabolic and anorexigenic signals. However, these changes should not be interpreted as evidence that GLP‐1 suppression directly mediates cocaine‐induced appetite suppression, since cocaine‐related appetite and weight effects are likely multifactorial.

In addition, a small open‐label case series reported heterogeneous but partially encouraging findings following repeated extended‐release exenatide in individuals with CUD [35]. While this exploratory study provided important preliminary data, its small sample size, lack of control group, and limited statistical power preclude definitive conclusions regarding treatment efficacy. Moreover, heterogeneity in patient characteristics and outcome measures further restricts the generalizability of its findings.

Findings from other addictive disorders, particularly alcohol use disorder, support the broader relevance of GLP‐1RA in addiction‐related reward processes, but their translation to CUD remains indirect and substance‐specific [40].

The potential development of GLP‐1RA in CUD therefore requires rigorously designed clinical trials to assess efficacy, safety, optimal dosing, and appropriate patients' selection. Available human data do not yet allow reliable predictors of response to be identified; however, future studies should prospectively assess candidate moderators, including BMI and metabolic status, baseline appetitive hormone profiles, cocaine use severity and pattern, craving intensity, treatment‐seeking status, co‐occurring alcohol or other substance use, psychiatric comorbidities, concomitant addiction care, and GLP‐1RA dose, duration, and formulation. Such trials should incorporate objective measures of cocaine use, validated craving and behavioral scales, and systematic monitoring of adverse events and potential misuse patterns.

Pending the availability of robust clinical data, off‐label use of GLP‐1RA for the treatment of CUD should be discouraged. Pharmacovigilance and addictovigilance systems should continue to monitor evolving patterns of use within this population.

In conclusion, GLP‐1 receptor signaling represents a biologically plausible and mechanistically supported target for CUD. However, current clinical evidence remains insufficient to support routine therapeutic use [40]. Several relevant questions require further investigation, including the optimal dosing strategies, duration of treatment, effects of treatment discontinuation, individual predictors of response, underlying mechanisms of action, interactions with psychiatric and medical comorbidities, and broader considerations related to cost and equitable access [41].

A dedicated clinical trial evaluating semaglutide for CUD is currently underway (ClinicalTrials.gov identifier: NCT07227948), which may provide more definitive data regarding efficacy and safety in this population [42].

## Author Contributions

**Céline Eiden:** conceptualization, supervision, data interpretation, original draft writing. **Ghjulia Chautard:** data collection, case analysis, figure and table writing. **Anne Batisse:** data collection, pharmacological interpretation, critical revision. **Aurélie Aquizerate:** data collection, pharmacological interpretation, critical revision. **Amandine Luquiens:** clinical interpretation, critical revision. **Hélène Donnadieu:** clinical interpretation, critical revision. **Jean‐Luc Faillie:** methodology, pharmacological interpretation, critical revision. **Hélène Peyriere:** supervision, conceptualization, methodology, critical revision.

## Funding

The authors have nothing to report.

## Ethics Statement

This study was conducted using anonymized data collected through the French national pharmacovigilance and addictovigilance systems as part of routine regulatory activities. No additional ethical approval was required.

## Consent

Patient data were anonymized prior to analysis in accordance with applicable regulations. According to applicable national procedures, individual informed consent was not required.

## Conflicts of Interest

The authors declare no conflicts of interest.

## Permission to Reproduce Material

Not applicable.

## Artificial Intelligence Use Statement

The authors used ChatGPT (OpenAI) for language editing and formatting assistance. All scientific content, interpretation, and conclusions were independently developed and verified by the authors.

## Data Availability

The data supporting the findings of this study are available from the corresponding author upon reasonable request, in accordance with regulatory restrictions related to vigilance data.
